# Supplementary material for: Molecular characterization of HAMP rs10421768 gene and phenotypic expression of hepcidin; a case-control study among sickle cell anaemia patients in Ghana
Source: PLoS One. 2024 Jun 27;19(6):e0306194. doi: 10.1371/journal.pone.0306194 (PMC11210777; doi:10.1371/journal.pone.0306194)
Supplement: Attachment — Submitted filename: Response to Reviewers.docx [file pone.0306194.s002.docx]

Dear Editor,

Thank you for your email. I am pleased to resubmit manuscript titled “**Molecular characterization of HAMP rs10421768 gene and phenotypic expression of hepcidin; a case-control study among sickle cell anaemia patients in Ghana**” for your consideration.

Authors do appreciate your endless efforts and rich comments to ensure improvement in the manuscript.

The concerns raised by the editorial team have been addressed and highlighted below and marked in the respective sections in the manuscript. I look forward to your favourable response.

Thank you.

**Reviewers' Comments:**

Reviewer #1:

i. The sample size is very limited, and the control cases are not enough for a comparable controlled study. More cases are required for proper statistical analysis.

Response: This was part of student academic thesis without any external financial support making it difficult to recruit larger sample size. This has been duly acknowledged as limitation

ii. Not using real-time PCR for SNP analysis (which the most common method in most of the similar researches) are not justified.

Response: Genotyping was performed by real-time polymerase chain reaction (PCR) using the Agena MassARRAY method with iPLEX® PCR (Inqaba Biotec^TM^ West Africa Ltd, Ghana) as stated in trhe methodology (line 187-192)

iii. The consent for the patients methodology is not clear.

Response: This has been revised for clarity and it reads “Participants 18years and above provided written informed consent. The consents of the study participants below 18years were obtained from guardians or parents“

Reviewer #2:

i. Conclusions are not entirely supported by the data.
   The relevance of the study lies in determining whether this polymorphism implies a higher risk of developing iron overload due to decreased hepcidin synthesis. Therefore, it would be very enriching if the authors conducted a measure of iron overload, such as measuring serum ferritin concentrations, for instance.

Response: The authors acknowledge your concerns. However, the study could not assess the iron status/profile of the participants, and this has been acknowledged as a limitation of the study

ii. It is important to divide the participants according to the different polymorphisms and compare the serum hepcidin concentrations to verify whether the polymorphism is indeed determining the hepcidin levels.

Response: This information is presented in figure 3. Only one participant had homozygous GG variant making it statistically inappropriate to separate it.

iii. Considering that the study did not establish any relationship between ferritin concentrations and the mapped polymorphisms, and that the objective was to evaluate the distribution of the polymorphisms, in this case, it is essential to present a sample size calculation.

Response: Determination of sample size is well presented in the methodology (Line 132-148)

iv. Lines 102-105: It doesn't seem appropriate to claim that the data from this study can provide appropriate guidance in managing heavily transfused individuals. I think it's important to downplay the significance. I believe the data can support future investigations. However, based on the applied methods and investigated variables, it's unclear whether the polymorphism would pose a higher risk of iron overload.

Response: this section has been revised “findings from this study would provide the possible relationship between the HAMP polymorphic variants and hepcidin protein expression in SCA patients”

v. Line 139: It is important to describe the criteria used to determine the severity of the disease.

Response: The detailed description of the severity score has been described in previous study by Hedo et al., 1993, and this has been referenced accordingly in the manuscript.

vi. Table 2: The ethnic-racial information of the participants was not provided. Please, provide it.

Response: Data regarding ethnicity were not captured. However, the study was conducted in Akan dominated population in Ghana

vii. Considering that the study's objective is to present the distribution of polymorphisms, it is important to have a more detailed description of the control group. Who are these participants? Are they from the same geographical region? Do they have similar socio-economic conditions? Why is the polymorphism distribution different in the control group? Please provide this information and discuss it.

Response: The control participants were people without sickle cell disease who reside in the same geographical area and of similar ethic group as the cases.

viii. Table 4: Please, you should exclude the written representation of microliter, etc

Response: this has been revised accordingly

ix. Lines 315-316: This comparison doesn't make much sense because the physiopathologies of the two diseases are completely different.

Response: This comparison has been expunged from the discussion

x. Lines 327-328: Iron overload increases hepcidin. It is not possible to compare with studies that do not separate individuals based on the presence or absence of iron overload. Or at least, a consideration should be made regarding this aspect.

Response: the comparison is on hepcidin levels and not iron. It is true iron overload increases hepcidin levels. It expression can also be inhibited by intense erythropoietic stress or hypoxia.

xi. Lines 337-339: please revise this sentence “…and the possible iron overload”.
    The discussion should address the limitations of the study.

Response: The phrase “…and the possible iron overload” has been expunged
